# Robust Automated Tumour Segmentation on Histological and Immunohistochemical Tissue Images

**DOI:** 10.1371/journal.pone.0015818

**Published:** 2011-02-28

**Authors:** Ching-Wei Wang

**Affiliations:** Graduate Institute of Biomedical Engineering, National Taiwan University of Science and Technology, Taipei City, Taiwan; National Microelectronics Center, Spain

## Abstract

Tissue microarray (TMA) is a high throughput analysis tool to identify new diagnostic and prognostic markers in human cancers. However, standard automated method in tumour detection on both routine histochemical and immunohistochemistry (IHC) images is under developed. This paper presents a robust automated tumour cell segmentation model which can be applied to both routine histochemical tissue slides and IHC slides and deal with finer pixel-based segmentation in comparison with blob or area based segmentation by existing approaches. The presented technique greatly improves the process of TMA construction and plays an important role in automated IHC quantification in biomarker analysis where excluding stroma areas is critical. With the finest pixel-based evaluation (instead of area-based or object-based), the experimental results show that the proposed method is able to achieve 80% accuracy and 78% accuracy in two different types of pathological virtual slides, i.e., routine histochemical H&E and IHC images, respectively. The presented technique greatly reduces labor-intensive workloads for pathologists and highly speeds up the process of TMA construction and provides a possibility for fully automated IHC quantification.

## Introduction

Tissue microarray (TMA) is an effective tool for high throughput molecular analysis to help identify new diagnostic and prognostic markers and targets in human cancers. The technique allows rapid visualization of molecular targets in thousands of tissue specimens at a time and facilitates rapid translation of molecular discoveries to clinical applications; it has been applied to the study of tumour biology, the development of diagnostic tests, the investigation of novel molecular biomarkers, laboratory quality assurance, and an excellent validation and translation platform for other types of high-throughput molecular research [1], [2], [3], [4].

**Figure 1:**
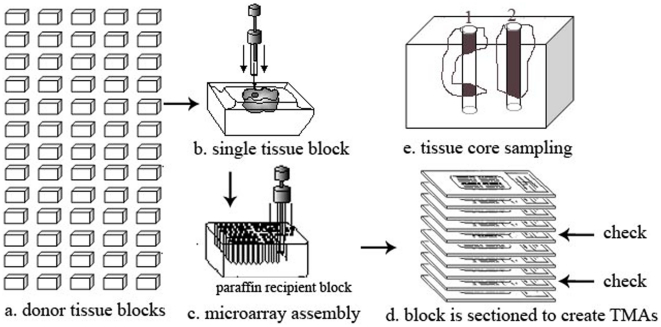
Tissue Microarray Construction. a. donor tissue blocks are selected, b. a needle is used to sample multiple cylindric tissue cores from each donor block and the sampling locations are carefully chosen by an experienced pathologist based on the top H&E slide of the block, c. the obtained tissue cores are assembled in a single microarray, d. the finished tissue microarray block is sectioned to create multiple TMAs where periodically a TMA slide is stained with H&E with all tissue cores examined by an experienced pathologist to verify if cancerous cells exist, e. tumour is with irregular shape and size; sections of cylindric tissue cores may not contain cancerous cells.

TMAs are produced by a method of re-locating tissue from histologic paraffin blocks such that tissue from multiple patients can be studied on the same slide (commonly, three to five tissue cores are extracted from each donor block). This is done by using a needle to biopsy a standard histologic sections and placing the core into an array on a recipient paraffin block (Fig. 1a,b,c), using a tissue microarrayer. The new block is then cut into 4-micron or 5-micron thick sections that contain 40 to hundreds tissue specimens (Fig. 1d), and these sections can then be stained using standard laboratory methods such as immunohistochemistry for various biomarker studies. In constructing TMAs, the location to sample each tissue core from individual donor blocks is carefully selected by an experienced pathologist at a region containing large amounts of cancer cells of the top H&E section. Tumour is a 3D object and has irregular shape, and thus the obtained cylindrical specimens (tissue cores) may not contain cancerous cell for all TMA sections; as illustrated in Fig. 1e, the tissue core 1 in a number of TMA sections derived from the middle of the cylindric specimens does not contain cancerous cell. In addition, it is unpredictable how deep the tumour is. Hence, periodically TMA slides are stained with H&E and pathologists have to visually examine all the tissue cores across TMAs (Fig. 1d), which is an extremely time consuming and labor-intensive process.

Immunohistochemistry (IHC) is widely used in investigation of novel molecular biomarkers. The conventional approach for protein expression quantification is for two pathologists to independently score all tissue cores across all TMAs. However, manual scoring is expensive, time consuming and subjective. Moreover, the lengthy pathologist-based scoring process has become the major bottleneck for this high throughput technique. Hence, the demand for robust and reliable automated quantification has become paramount. A technical challenge of quantifying protein expression is that the measurement is required to be conducted on the cancerous cells only. Existing research [5], [6], [7], [8] on IHC quantification make simplification to the measurement problem by assuming the knowledge of tumour areas and requires manual segmentation of tumour cells.

Computer-assisted image analysis of IHC has been shown to reduce the variation in analysis of staining levels [9]. A variety of studies have been published exploring the use of image analysis and machine vision for tissue analysis and biomarker measurement [5], [10]. Camp et al. [10] have proposed a system called AQUA for quantification of biomarker expression based on FISH where specific fluorescent stains can be used for cell compartmentalization to detect nuclei, cytoplasm and membranes [11]. Robust automated approaches for IHC quantification are still under-developed and require the empirical evaluation of algorithms which can both measure the intensity and distribution of biomarker, but also do this within the architectural components of the tissue sample that are relevant to the study.

As a result, the aim of this study is to develop an automated cancerous cell segmentation method in both routine histochemical H&E and IHC tissue images. Karacali and Tozeren [12] presented an automated method to detect regions of interest in whole slide H&E breast tissue slides for sampling tissue cores. However, the method is for classification on large image blocks and does not deal with small tissue cores or IHC images. In addition, the breast tissue images used in [12] show distinctive blue and red/pink stains in their Hematoxylin and Eosin (H&E) images, which however do not apply to the lung tissue specimens we used. In comparison, the lung tissue images in our experiments appear low contrast features with red/pink stains, which makes tumour detection more challenging.

In this paper, a robust tumour segmentation technique is developed and tested on the two commonly used pathological data, including routine H&E and IHC virtual slides. The method includes a tissue architecture extraction approach and a tumour texture learning model; the tissue architecture extraction approach contains a stain separation method and an a unsupervised multistage entropy-based segmentation method, and the tumour texture learning is an MRF image segmentation system. The method allows fine pixel based segmentation for small tissue cores, and three classes of tissue morphology were defined, including tumour, stroma and lymphoid/inflammatory cells/necrosis.

In experiments, we tested the method on two types of data, including nine H&E lung tissue virtual slides and nine IHC slides stained with BAX [13]. In evaluation, although many researchers use object-based quantitative evaluation (as long as k% of the object is accurately classified where k% can be set as 50%, it is counted as correct), the object-based quantitative evaluation allows pixel-based misclassification and tends to show better performance results than real performance outcomes. Here, a much more strict pixel-based quantitative evaluation was conducted by automatically comparing the system outputs with the manually segmented ground truth data. The experimental results show that the presented system achieves 80% and 78% accuracy for pixel-based segmentation in H&E and IHC respectively.

The outline of this paper is as follows. The automated tumour detection method is introduced in section 0, and the experimental results are displayed in section with quantitative performance evaluation. The paper is concluded in section 0.4.

## Methods

**Figure 2:**
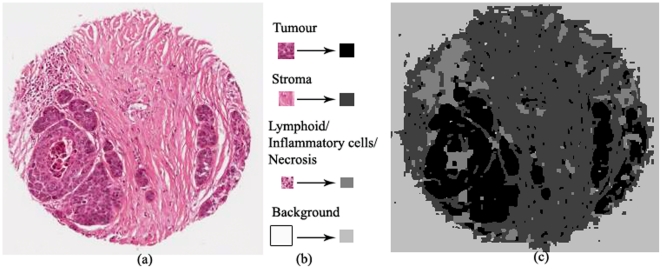
Supervised cell segmentation on (a) a H&E lung tissue core image, (b) four classes of tissue morphologies are defined for supervised learning, including “tumour”, “stroma”, “lymphoid/inflammatory cells/necrosis” and “background”, (c) the segmentation result.

The intelligent tumour segmentation system contains a tissue architecture extraction model and a tumour texture modelling method based on the extracted tissue architecture patterns. The tumour texture modelling method is based on a Markov Random Field image segmentation model [14], and the theoretical framework relies on Bayesian estimation via combinatorial optimization (Metropolis algorithm/simulated annealing). The final segmentation is obtained by classifying the pixels into different pixel classes. In this work, four classes with similar tissue morphology were defined, including tumour, stroma, lymphoid/inflammatory cells/necrosis and background (see Fig. 2), and regions of individual classes were manually selected for supervised learning.

### 0.1 Tissue Architecture Extraction

#### 1. Stain separation

The Lambert-Beer's law describes an exponential relationship between the intensity of monochromatic light transmitted through a specimen and the amount of stain present in the specimen:
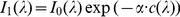
(1)where 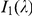 is the intensity of light of wavelength 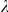 transmitted through the specimen (the intensity of light detected), 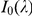 is the intensity of light of wavelength 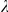 entering the specimen, 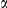 is the amount of stain per unit area of the specimen, and 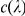 is a wavelength-dependent factor reflecting the absorption characteristics of the particular stain.

The CCD RGB cameras use three broad-band filters to capture color images in three channels. As the relative intensity 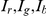 in each of the RGB channels depends on the concentration of stain in a nonlinear way [15], the intensity values of the image can not directly be used for separation and measurement of each of the stains, but the optical density (OD) for each channel can be defined as
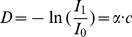
(2)


The OD for each channel is linear with the amount of stain, given the absorption value, and can therefore be used for extracting the amount of stain in a specimen. Each stain can be characterized by a specific OD for the light in each of the three RGB channels, which can be represented by a 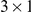 OD vector describing the stain in the OD-converted RGB color space [16]. Hence, in the case of two stains, the color system can be described as
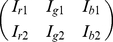
(3)where each row represents a specific stain and each column represents the OD as detected by RGB channels for individual stain.

Color deconvolution [16] can be used to obtain independent information about each stain's contribution based on orthonormal transformation of the RGB information, and the transformation has to be normalized to achieve correct balancing of the absorbtion factor for separate stains. For normalization, each OD vector is divided by its total length to obtain a normalized OD array 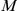. If 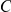 is the 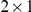 vector for amounts of the two stains at a particular pixel, then the vector of OD levels detected at that pixel is 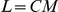. Defining 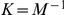 as the color-deconvolution array, we can therefore obtain individual stain information by 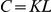.

For example, given an IHC image, we first separate independent DAB and Haematoxylin stain contributions by the color deconvolution approach [16]. In this study, the normalized optical density (OD) matrix, 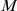, to describe the colour system for orthonormal transformation is defined as follows:
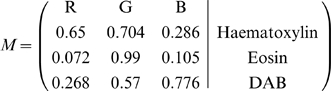
(4)


Given 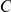 is 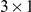 vector for amounts of the three stains at a particular pixel, the vector of OD levels detected at that pixel is equal to 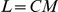. Therefore, multiplication of the OD image with the inverse of OD matrix results in orthogonal representation of the stains forming the image (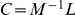), and hence colour de-convolution matrix is defined as:
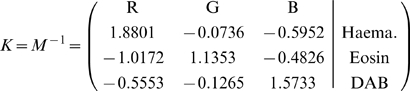
(5)


The extracted Haematoxylin OD image is applied with the multistage entropy-based segmentation method to extract tissue nuclear architecture information.

#### 2. Multistage Entropy-Based Segmentation of Nuclear Architecture

All statistical operations are performed on the normalized image histogram, 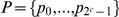 where the valid intensity scales from 0 to 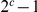, and image entropy E(P) is calculated using discrete histogram P as follows.
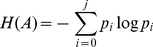
(6)

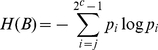
(7)

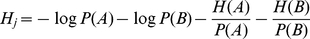
(8)where 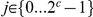, 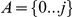 and 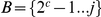.

The entropy maximum is calculated as 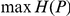, which defines the cut-off point 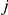 for assigning image pixels into different classes where 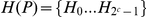.

After calculating 2D image histogram entropy function, we first apply an eight stage maximum entropy function to automatically separate input image into eight layers, and then a two stage entropy function to extract potential regions of nuclei, which is then processed by morphological operations to produce final nuclear segmentation results. The algorithm is described below.

- divide histogram into four equal sub-histograms 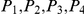, obtaining 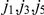 where 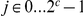

- compute maximum entropy points 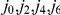 for the four different 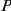 intervals, where 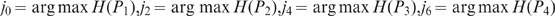

- use 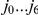 to categorize input image into eight layers
- calculate new histogram 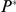

- compute 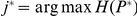 and categorize input image into 2 categories, including nuclei and non-nuclei
- apply the morphological operations described below

The purpose of the morphological function is both to reduce spurious false positive detection and increase low contrast true negative detection. The method re-assigns each image pixel value using the most frequent intensity level within its neighborhood. Given an image 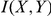 and neighborhood radius 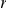, the output image 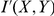 is formulated as follows.
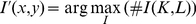
(9)where 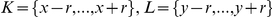, and r is empirically set as 3.

### 0.2. Tumour Texture Learning and Segmentation

#### 1. Texture Feature Extraction

The H&E staining method colors nuclei of cells blue by Hematoxylin, and the nuclear staining is followed by counter-staining with Eosin, which colors other structures in various shades of red and pink. Regarding bright field immunohistochemistry staining method, Hematoxylin induces blue staining of nuclei and DAB induces brown staining (protein expression) of various cell compartments. In our previous study [17], we discovered that the blue channel had higher discriminative information in the classification of two types of Non Small Cell Lung Carcinomas using H&E tissue images than composite greyscale, red and green channels. In addition, the morphology of nuclei is used as a common indication of cancerous cells. Hence, the blue channel information is extracted as image features for subsequent tumour cell segmentation.

Given a set of sites 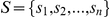 of an image and the corresponding set of image observation 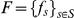, attributes of each class 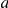 to learn in the training set include the mean 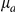 and variance 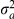 of 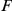. The learned class attributes were then sent to MRF image segmentation model to find MAP estimation.
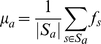
(10)

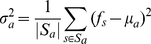
(11)where 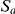 denotes the set of pixels in the training set of class 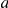 and 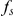 is the image observation value at pixel 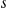.

#### 2. MRF Segmentation

Given a set of sites 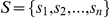 and a set of image data 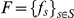, each site belongs to any one of four classes defined. A global discrete labelling 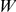 assigns one label 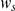 to each site 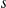 in 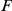. Thus, the pair 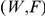 specifies a segmentation. To find the optimal labelling 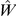 with maximum the posterior probability 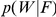, using independence assumption [18] and Bayesian theorem 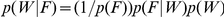, 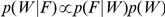. Thus, the MAP estimation (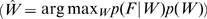) is given by
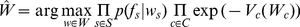
(12)where 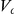 denotes the clique potential of clique 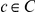 having the label configuration 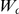. Assuming that 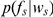 is Gaussian, the energy function 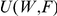 is given by
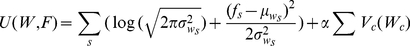
(13)


The local energy of any labeling 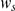 is: 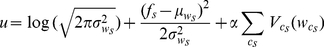
(14)where 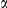 is the weight of the prior term and is experimentally set as 0.9.

The problem is reduced to a combinatorial optimization problem, that is to minimizing a non-convex energy function 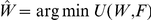. Each clique corresponds to a pair of neighboring pixels, and the clique potential is designed to favor similar classes in neighboring pixels.
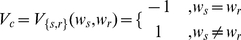
(15)


According to Hammersley-Clifford theorem [19], 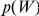 follows a Gibbs distribution, 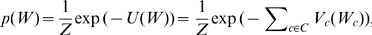, where 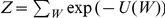 is the partition function. Therefore, the full prior is:
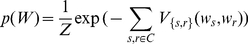
(16)


The estimation of 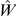 is then computed through the energy minimization using a relaxation method. Four methods were tested initially, including Metropolis algorithm [20], Modified Metropolis algorithm (MMD) [21], Iterated Conditional Mode (ICM) [18] and Gibbs sampling [19], and the preliminary experiments show that Metropolis algorithm obtains best segmentation results and is thus used in the experimental section.

#### 3. Parameter Definition

The number of pixel classes is defined as four, including “tumour”, “stroma”, “lymphoid/inflammatory cells/necrosis” and “background” (see Fig. 2). Although the aim of the study is to separate cancerous cells from other cells and background where only three classes (“tumour”, “non-tumour”, “background”) are needed, our preliminary exploration showed that tumour cell detection performs better when the non-tumour class is further divided into two classes (“stroma” and “lymphoid/inflammatory cells/necrosis”) as these two subtypes have distinctively different morphology. A training set was obtained for supervised learning by manually selecting representative regions on the input image. In supervised image segmentation, the mean 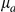 and standard deviation 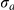 of each class was computed from the training set. After MRF image segmentation described in the previous sections, pixels assigned to the two non-tumour subtypes were merged into one non-tumour class for evaluation in the next section.

## Results

The presented tumour cell detection system is evaluated with nine H&E tissue core images of lung carcinoma and nine bright field immunohistochemistry tissue core images of lung carcinoma with a biomarker named BAX [13]. Regarding the image dimension, it is 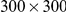 for H&E tissue cores on average and 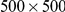 for immunohistochemistry tissue cores. For quantitative performance evaluation, a ground truth dataset was produced by independent manually marking. As for immunohistochemistry, some regions such as poorly differentiated cases can be difficult for even an experienced pathologist to define whether they are cancerous cells or not. In evaluation, the pixels of these regions are excluded.

The outputs by the presented method were then compared with the ground truth data to generate confusion matrices [22] and other performance indices were generated, including accuracy, true positive rate, true negative rate, false positive rate, false negative rate and precision for quantitative performance evaluation on cancerous cell segmentation.

### 0.3. Histological Slides: H&E

**Figure 3:**
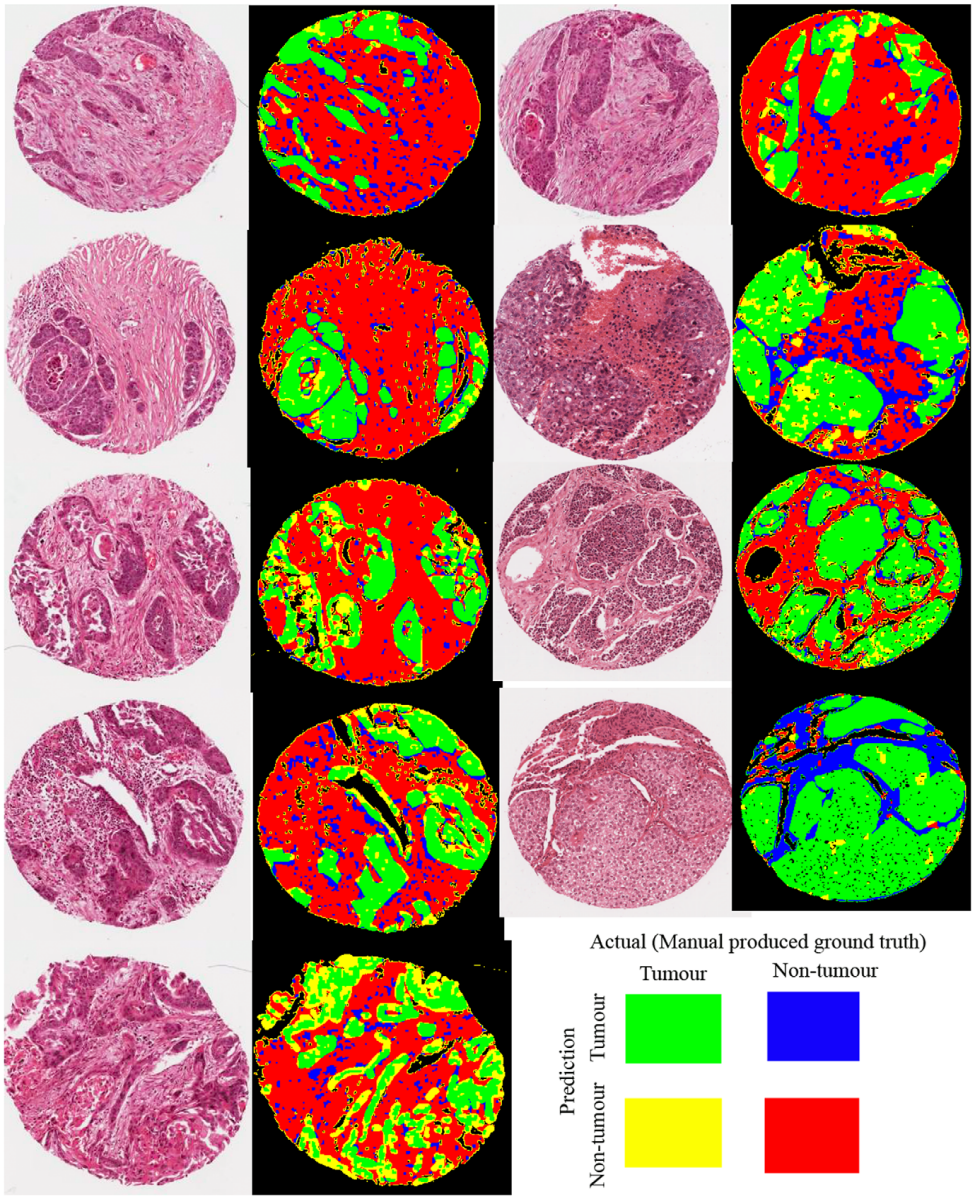
Evaluation outputs of tumour detection on routine H&E. green = TP, red = TN, yellow = false negative, and blue = false positive.

The quantitative results are shown in Table 1. Overall, the presented system achieves 80% accuracy and 79% precision in pixel based cancerous cell segmentation, and the image outputs based on the evaluation results are displayed in Fig. 3, showing that the technique is able to in identify cancerous cell on low contrast H&E lung tissue core images.

**Table 1:** Pixel-based quantitative evaluation on tumour detection results in H&E tissue images.

	Accuracy	TP rate	FP rate	FN rate	TN rate	Precision
1	0.84	0.84	0.18	0.16	0.82	0.92
2	0.8	0.78	0.17	0.22	0.83	0.87
3	0.74	0.95	0.88	0.05	0.12	0.76
4	0.84	0.81	0.15	0.19	0.85	0.62
5	0.84	0.71	0.11	0.29	0.89	0.71
6	0.85	0.71	0.09	0.29	0.91	0.78
7	0.82	0.62	0.06	0.38	0.94	0.86
8	0.77	0.66	0.16	0.34	0.84	0.73
9	0.71	0.49	0.1	0.51	0.9	0.81
Aver.	0.8	0.73	0.21	0.27	0.79	0.79

TP: number of true positive pixels; TN: number of true negative pixels; FP: number of false positive pixels; FN: number of false negative pixels; Accuracy = (TP+TN)/(TP+TN+FP+FN); TP rate = TP/(TP+FN); FP rate = FP/(FP+TP); FN rate = FN/(FN+TP); Precision = TP/(TP+FP).

### 0.4 Bright Field Immunohistochemistry

**Figure 4:**
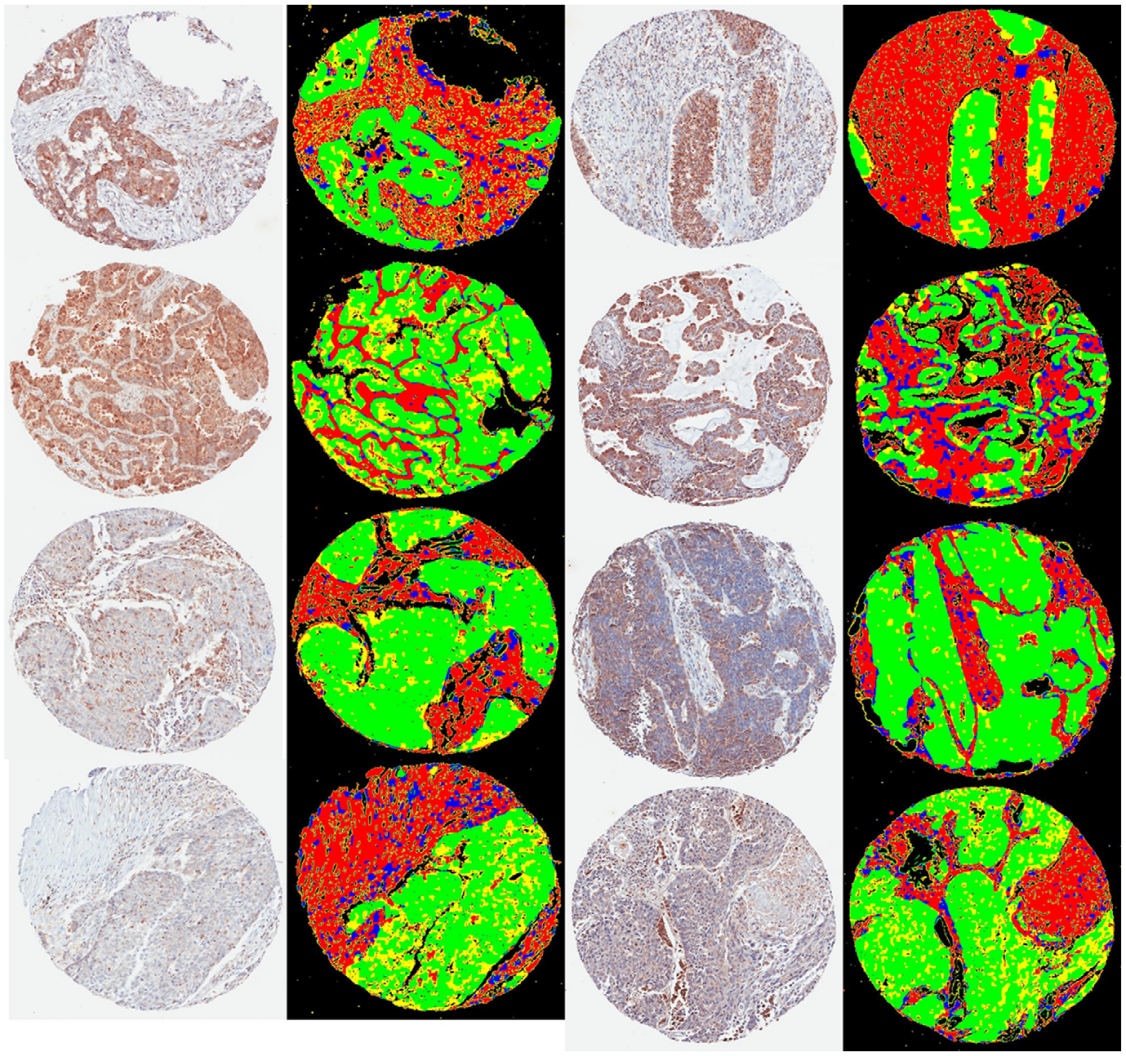
Evaluation of Tumour Detection on IHC Image. green = TP, red = TN, yellow = false negative, and blue = false positive.

The quantitative results are shown in Table 2. Overall, the presented system achieves 78% accuracy and 89% precision in pixel based cancerous cell segmentation, and the image outputs based on the evaluation results are displayed in Fig. 4. The results show that the technique is able to identify cancerous cell on immunohistochemistry tissue core images.

**Table 2:** Pixel-based quantitative evaluation on tumour detection results in Immunohistochemistry tissue images.

	Accuracy	TP rate	FP rate	FN rate	TN rate	Precision
1	0.79	0.7	0.09	0.3	0.91	0.91
2	0.93	0.73	0.01	0.27	0.99	0.94
3	0.75	0.71	0.14	0.29	0.86	0.94
4	0.79	0.77	0.14	0.23	0.86	0.94
5	0.68	0.57	0.17	0.43	0.83	0.83
6	0.82	0.51	0.03	0.49	0.97	0.89
7	0.73	0.66	0.21	0.34	0.79	0.75
8	0.85	0.88	0.23	0.12	0.77	0.91
9	0.7	0.65	0.18	0.35	0.82	0.91
Aver.	0.78	0.69	0.13	0.31	0.87	0.89

TP: number of true positive pixels; TN: number of true negative pixels; FP: number of false positive pixels; FN: number of false negative pixels; Accuracy = (TP+TN)/(TP+TN+FP+FN); TP rate = TP/(TP+FN); FP rate = FP/(FP+TP); FN rate = FN/(FN+TP); Precision = TP/(TP+FP).

## Discussion

We have demonstrated an automated technique to automatically segment cancerous cells, for TMA construction and IHC quantification, on lung tissue images. The supervised image segmentation system includes a feature extraction function and an MRF based Bayesian estimation method for modelling four types of texture based on the tissue morphology defined. The system is demonstrated to be able to identify cancerous cells and achieve 80% accuracy and 79% precision on routine histochemical images and 78% accuracy and 89% precision on IHC images, based on pixel based evaluation results. The presented technique greatly reduces the workload of pathologists, speeds up the process of TMA construction and provides a possibility for fully automated IHC quantification. In future work, we plan to improve the technique by enhance the simple tumour texture feature extraction process and further apply the technique to different IHC slides. Moreover, we would like to develop a fully automated biomarker quantification system based on the outputs of this method.
