# Correction: Robust Automated Tumour Segmentation on Histological and Immunohistochemical Tissue Images

**DOI:** 10.1371/annotation/ee80660c-98ef-477b-bc56-d81246acd7c5

**Published:** 2011-04-11

**Authors:** Ching-Wei Wang

Ching-Wei Wang would like to add four authors to the byline. The updated byline is: Ching-Wei Wang 1, Dean Fennell 2, Ian Paul 2, Kienan Savage 2, Peter Hamilton 2

1Graduate Institute of Biomedical Engineering, National Taiwan University of Science and Technology, Taipei City, Taiwan, 2 Centre for Cancer Research and Cell Biology, Queens University Belfast, Belfast, United Kingdom

The new citation is: Wang C-W, Fennell D, Paul I, Savage K, Hamilton P (2011) Robust Automated Tumour Segmentation on Histological and Immunohistochemical Tissue Images. PLoS ONE 6(2): e15818. doi:10.1371/journal.pone.0015818

Competing Interest: Peter Hamilton is founder and shareholder in i-Path Diagnostics Ltd.

All new authors contributed reagents/materials/analysis tools 

